# Bridging the gap between science and policy: an international survey of scientists and policy makers in China and Canada

**DOI:** 10.1186/s13012-016-0377-7

**Published:** 2016-02-06

**Authors:** Bernard C. K. Choi, Liping Li, Yaogui Lu, Li R. Zhang, Yao Zhu, Anita W. P. Pak, Yue Chen, Julian Little

**Affiliations:** 1 Injury Prevention Research Centre, Medical College of Shantou University, Shantou, China; 2 School of Epidemiology, Public Health and Preventive Medicine, University of Ottawa, Ottawa, ON Canada; 3 Population and Public Health Program, British Columbia Provincial Health Services Authority, Vancouver, BC Canada; 4 Pak Consulting, Ottawa, ON Canada

**Keywords:** Knowledge transfer, Scientists, Policy makers, Public health, International study

## Abstract

**Background:**

Bridging the gap between science and policy is an important task in evidence-informed policy making. The objective of this study is to prioritize ways to bridge the gap.

**Methods:**

The study was based on an online survey of high-ranking scientists and policy makers who have a senior position in universities and governments in the health sector in China and Canada. The sampling frame comprised of universities with schools of public health and medicine and various levels of government in health and public health. Participants included university presidents and professors, and government deputy ministers, directors general and directors working in the health field. Fourteen strategies were presented to the participants for ranking as current ways and ideal ways in the future to bridge the gap between science and policy.

**Results:**

Over a 3-month survey period, there were 121 participants in China and 86 in Canada with response rates of 30.0 and 15.9 %, respectively. The top strategies selected by respondents included focus on policy (conducting research that focuses on policy questions), science-policy forums, and policy briefs, both as current ways and ideal ways to bridge the gap between science and policy. Conferences were considered a priority strategy as a current way, but not an ideal way in the future. Canadian participants were more in favor of using information technology (web-based portals and email updates) than their Chinese counterparts. Among Canadian participants, two strategies that were ranked low as current ways (collaboration in study design and collaboration in analysis) became a priority as ideal ways. This could signal a change in thinking in shifting the focus from the “back end” or “downstream” (knowledge dissemination) of the knowledge transfer process to the “front end” or “upstream” (knowledge generation).

**Conclusions:**

Our international study has confirmed a number of previously reported priority strategies to bridge the gap between science and policy. More importantly, our study has contributed to the future work on evidence-based policy making by comparing the responses from China and Canada and the current and ideal way for the future. Our study shows that the concept and strategies of bridging the gap between science and policy are not static but varying in space and evolving over time.

## Background

“Evidence-informed policy making” is an interactive process that involves effective exchanges of knowledge between scientific evidence producers (scientists) and scientific evidence users (policy makers) [1, 2]. To facilitate this process, finding new and effective ways to bridge the gap between science and policy becomes an important task [3, 4].

The objective of this study is to explore and prioritize ways to bridge the gap between science and policy in the health fields. It is an international comparison study of China and Canada on how research findings are currently being used and can best be promoted in the future for policy making and development and evaluation of programs and practice.

The study was conducted in China and Canada because there is a wide difference in the history, culture, and way of thinking. This can maximize the possibility of finding new ideas and learning from the East and the West. Healthcare provided under social insurance systems vary in these two countries, with marked differences in access by geographical location and affluence. There are challenges of urban concentrations and sparsely occupied rural regions, with very large geographical areas of coverage. There are considerable population movements, in China from rural to urban areas, and in Canada because of immigration. Education and training of scientists and policy makers in China and Canada are very different, leading to different perspectives and philosophies. A comparison study conducted in Canada and the USA, for example, might be less fruitful due to their similarities in many aspects.

The study was conducted among university and government scientists/officials that were considered high-ranking (having a senior position) based on their job titles. This increases the possibility of the study to include participants who are likely to be involved in the evidence-informed policy-making process.

## Methods

The study was based on an online survey conducted in 2012 (offered in English, French, and Chinese) of high-ranking senior position scientists and policy makers in health, medicine, and public health in universities and governments in China and Canada. The study protocol was pre-approved by the research ethics boards of the Medical College of Shantou University, China and the Faculty of Medicine of University of Ottawa, Canada.

### Survey instrument

A literature review conducted in 2010 by two authors (BC and LZ) identified 23 key strategies for bridging the gap between science and policy. The search was conducted by a reference librarian to identify peer-reviewed journal articles addressing ways to improve knowledge exchange in health policies. The search covered five databases: MEDLINE, EMBASE, Global Health, PsycINFO, and Social Policy and Practice. No constraint was put on publication date, but searches were limited to the English language. The review included only studies that actively collected data by means of surveys, focus groups, and/or key informant consultations, and excluded review articles, opinion pieces, studies without a data collection component, studies that evaluated the implementation of one specific strategy to bridge the gap between science and policy, and studies that restricted their discussions to theoretical knowledge exchange frameworks. Of the 912 records identified from the 5 databases, 10 articles published between 2002 and 2009 satisfied all inclusion criteria [5–14]. As we anticipated that the participants (senior researchers and officials) would have limited time available to complete the survey, the 23 strategies were further combined and reduced to 14 (Table 1). This was done by expert review for overlap by the two authors, with differences resolved by a consensus process. The participants were asked to indicate the top 5 strategies out of these 14 strategies that they considered current ways, and ideal ways in the future, to bridge the gap between science and policy.

**Table 1:** Fourteen strategies to bridge the gap between science and policy used in the China-Canada survey 2012

Code	Short title	Strategy
a	Collaboration in study design	Involvement of policy makers in the design and framing of research projects.
b	Focus on policy	Conduct of research that focuses on policy questions.
c	Policy briefs	Creative and good packaging of research findings for policy makers – policy briefs, synthesis and summaries, systematic reviews, etc.
d	Web-based portals	Web-based portal/inventory for access to evidence for policy making.
e	Email updates	Email updates of new research or summaries of current research to policy makers.
f	Journal publications	Publications in peer-reviewed journals.
g	Conferences	Conferences and meetings.
h	Policy recommendations	Development of explicit policy recommendations or summaries for research findings.
i	Science-policy forums	Forums for researchers and policy makers to present and hear about research findings and policy requirements.
j	Joint research projects	Partnerships between university scientists and government scientists in joint research projects.
k	Personal contact	Personal contact between scientists and policy makers.
l	Knowledge brokers	Utilization of third party knowledge brokers (information specialists or consultants) to go between scientists and policy makers.
m	Collaboration in analysis	Collaboration between scientists and policy makers in analysis, writing up, and/or dissemination of findings.
n	Co-authorship	Co-authorship of a research publication between scientists and policy makers.

The questionnaire was developed in English, and then translated into French and Chinese, together with the cover letter, invitation, and reminders. Each translation was reviewed independently by at least another translation expert to ensure accuracy. The questionnaires in three languages were uploaded onto the online survey system “FluidSurveys”. The questionnaires were pretested online by a small number of potential participants whose responses were excluded from the analysis. The final questionnaire was organized into four sections, comprising multiple-choice questions on: (1) demographic characteristics of the participants, (2) perception of the importance of bridging the gap between science and policy, (3) current ways being used to bridge the gap between science and policy (respondents were asked to indicate the top 5 out of 14 strategies), and (4) ideal ways that could be used in the future to bridge the gap between science and policy (top 5 out of 14 strategies).

### Survey participants

Survey participants included university and government scientists and officials in health and public health that were considered high-ranking (having a senior position) based on their job titles. The target population of the survey were presidents and professors of public health and medicine of universities, deputy ministers, directors general, and directors of health and public health of various levels of government in China and Canada. Sample size calculations using different percentage combinations, based on a 25 % difference in opinion between China and Canada, and an *α* error of 0.05 and *ß* error of 0.2, showed that a sample size of 70 respondents each for China and Canada was sufficient for the purpose of the study. To account for a possible response rate of 20 %, a minimum of 350 invited participants in each country were required.

In China, 16 randomly selected universities (out of 2236), the central government, 11 randomly selected provinces and regions (out of 34), and 50 randomly selected counties (out of 2862) were included in the sampling frame. In Canada, all universities (total of 98), the federal government, all provinces and territories (total of 13), and 30 randomly selected health regions (out of 84) were included in the sampling frame. In both countries, academic schools and departments of public health in universities, and central/federal government health departments, agencies, provincial health ministries and regional bureaus of health, public health, and disease control were included. Potential survey participants were identified from online university and government directories, and emailed enquiries to respective organizations, and were included if their job titles belonged to any of three categories: (1) university presidents and deputy ministers, (2) full/associate professors and directors general, or (3) assistant professors and directors. From all potential candidates meeting the above selection criteria, a total of 403 from China and 540 from Canada were invited to participate in the survey (Table 2). Job title selection was the only screening to increase the possibility of participants having involvement in the evidence-informed policy-making process. No attempt was made in the study to ensure that the individuals selected actually had some insight or experience related to evidence-informed policy.

**Table 2:** Distribution of invited participants, by subgroup and country, in the China-Canada survey 2012

Subgroup based on job title	China *N* = 403 (100 %)	Canada *N* = 540 (100 %)
University - level 1 (e.g., university presidents)	17 (4 %)	18 (3 %)
University - level 2 (e.g., full/associate professors)	119 (30 %)	87 (16 %)
University - level 3 (e.g., assistant professors)	37 (9 %)	66 (12 %)
Government - level 1 (e.g., deputy ministers)	64 (16 %)	127 (24 %)
Government - level 2 (e.g., directors general)	106 (26 %)	58 (11 %)
Government - level 3 (e.g., directors)	60 (15 %)	184 (34 %)

### Online survey

The online anonymous survey was conducted simultaneously in China and Canada from May 23 to August 29, 2012 (98 days). This period was considered a good time to conduct the survey based on consideration of annual work cycles (e.g., research grant deadlines, fiscal year end deadlines) within university and government sectors in both countries. All participants were given the option to complete the survey in their preferred language among English, French, or Chinese. No signed informed consent form was used as all responses were voluntary and completed responses provided implied consent. Those who did not complete the online survey were sent email reminders up to four times (on days 7, 21, 42, 70 since the initial invitation).

### Data analysis

Baseline characteristics of participants in the China-Canada survey were compared. Strategies (current and ideal) for bridging the gap between science and policy were ranked based on the percentage of respondents selecting each strategy as one of their top five, for China and Canada together and separately. Tests of significance were conducted using a *t* test for proportions. A difference of two proportions with a *p* value <0.05 (two-sided) was considered statistically significant.

## Results

From the invited participants in China (*N* = 403) and Canada (*N* = 540), 121 and 86 completed surveys were received from China and Canada, respectively. The response rates were significantly different, with China’s (30.0 %) almost twice that of Canada (15.9 %). It would have been useful to compare response rates in the six stratified subgroups (Table 2). However, this was not possible as the online survey was anonymous. We knew how many surveys were sent out in each subgroup, but we did not know how many people responded in each subgroup. In theory, we did identify the current roles of the respondents (Table 3) and therefore differences in subgroup-specific response rates could have been examined based on the self-reported roles. However, the self-reported roles of the respondents were often multiple and included both university and government, and many did not indicate their job title.

**Table 3:** Characteristics of participants in the China-Canada survey 2012

Characteristic/opinion	China * n* = 121 (100 %)	Canada * n* = 86 (100 %)	*t*	*p* value
1. Current role				
A scientist	69 (57 %)	25 (29 %)	3.98	*p* < 0.05*
A policy maker	23 (19 %)	26 (30 %)	−1.83	n.s.
Both a scientist and a policy maker	29 (24 %)	35 (41 %)	−2.60	*p* < 0.05*
2. Years working as a scientist				
Never	23 (19 %)	26 (30 %)	−1.83	n.s.
1–9 years	12 (10 %)	13 (15 %)	−1.09	n.s.
10–19 years	34 (28 %)	18 (21 %)	1.14	n.s.
20+ years	52 (43 %)	29 (34 %)	1.31	n.s.
3. Years working as a policy maker				
Never	69 (57 %)	25 (29 %)	3.98	*p* < 0.05*
1-9 years	24 (20 %)	17 (20 %)	0.00	n.s.
10–19 years	20 (17 %)	33 (38 %)	−3.41	*p* < 0.05*
20+ years	8 (7 %)	11 (13 %)	−1.45	n.s.
4. Sex				
Male	75 (62 %)	40 (47 %)	2.14	*p* < 0.05*
Female	46 (38 %)	46 (53 %)	−2.14	*p* < 0.05*
5. Age				
<35 years	11 (9 %)	1 (1 %)	2.45	*p* < 0.05*
35–54 years	94 (78 %)	44 (51 %)	4.06	*p* < 0.05*
55+ years	16 (13 %)	41 (48 %)	−5.56	*p* < 0.05*
6. How important do you think it is to bridge the gap between science and policy?				
Very important	78 (64 %)	68 (79 %)	−2.33	*p* < 0.05*
Somewhat important	36 (30 %)	14 (16 %)	2.32	*p* < 0.05*
Neither important nor unimportant	6 (5 %)	3 (3 %)	0.71	n.s.
Somewhat unimportant	1 (1 %)	0 (0 %)	0.93	n.s.
Very unimportant	0 (0 %)	1 (1 %)	−1.10	n.s.

*n.s.* non-significant

*p < 0.05 (two-sided), **p < 0.01 (two-sided)

Table 3 summarizes the characteristics of participants in the survey. In terms of current roles, significantly higher percentages of respondents in China than in Canada were scientists, while significantly fewer of them were both scientists and policy makers. There were no significant differences between Chinese and Canadian respondents in how long they have worked as scientists. However, compared to their Canadian colleagues, significantly fewer Chinese have worked as policy makers at all, or have worked for more than 10 years.

Almost two thirds of the respondents in China were male, whereas in Canada a more even distribution between males and females was observed. A much higher proportion of Chinese respondents (85 %) than their Canadian counterparts (52 %) were aged less than 55 years.

The majority of all respondents considered bridging the gap between science and policy to be “very important” or “somewhat important” (94 % in China, 95 % in Canada). Only one participant each in China and Canada considered bridging such gap to be “somewhat unimportant” or “very unimportant”.

The ranking of 14 strategies as *current* ways to bridge the gap between science and policy is shown in Table 4. The top three ranked current strategies selected by respondents in China and Canada were: (1) “b. Focus on policy” (57 %), (2) “i. Science-policy forums” (54 %), and (3) “c. Policy briefs” (47 %), and “g. Conferences” (47 %). The top three strategies selected by participants in China were: (1) “b. Focus on policy” (60 %), (2) “i. Science-policy forums” (54 %), and (3) “c. Policy briefs” (50 %). The top three strategies selected by those in Canada were: (1) “i. Science-policy forums” (55 %), (2) “b. Focus on policy” (51 %), and (3) “g. Conferences” (44 %). There were no significant differences for most of the current strategies between China and Canada, except for three strategies: “d. Web-based portals” and “f. Journal publications” were rated significantly lower by Chinese than by Canadians (17 vs. 30 % and 23 vs. 43 %, respectively), while “j. Joint research projects” was rated significantly higher by Chinese respondents than by Canadian ones (48 vs. 31 %).

**Table 4:** Ranking of 14 strategies by percentage in favor of each strategy with respect to the question “What are the current ways being used to bridge the gap between science and policy?” in the China-Canada survey 2012

Strategy	China and Canada *n* = 207 (100 %)	China *n* = 121 (100 %)	Canada *n* = 86 (100 %)	*p* value (China vs. Canada)
a. Collaboration in study design	No. 7, 77 (37 %)	No. 7, 51 (42 %)	No. 9, 26 (30 %)	n.s.
b. Focus on policy	No. 1, 117 (57 %)	No. 1, 73 (60 %)	No. 2, 44 (51 %)	n.s.
c. Policy briefs	No. 3, 97 (47 %)	No. 3, 61 (50 %)	No. 5, 36 (42 %)	n.s.
d. Web-based portals	No. 11, 46 (22 %)	No. 11, 20 (17 %)	No. 9, 26 (30 %)	*p* < 0.05*
e. Email updates	No. 13, 33 (16 %)	No. 13, 16 (13 %)	No. 13, 17 (20 %)	n.s.
f. Journal publications	No. 9, 65 (31 %)	No. 10, 28 (23 %)	No. 4, 37 (43 %)	*p* < 0.01**
g. Conferences	No. 3, 97 (47 %)	No. 4, 59 (49 %)	No. 3, 38 (44 %)	n.s.
h. Policy recommendations	No. 5, 85 (41 %)	No. 6, 56 (46 %)	No. 7, 29 (34 %)	n.s.
i. Science-policy forums	No. 2, 112 (54 %)	No. 2, 65 (54 %)	No. 1, 47 (55 %)	n.s.
j. Joint research projects	No. 5, 85 (41 %)	No. 5, 58 (48 %)	No. 8, 27 (31 %)	*p* < 0.05*
k. Personal contact	No. 8, 70 (34 %)	No. 9, 35 (29 %)	No. 6, 35 (41 %)	n.s.
l. Knowledge brokers	No. 12, 41 (20 %)	No. 12, 19 (16 %)	No. 11, 22 (26 %)	n.s.
m. Collaboration in analysis	No. 10, 61 (29 %)	No. 8, 40 (33 %)	No. 12, 21 (24 %)	n.s.
n. Co-authorship	No. 14, 8 (4 %)	No. 14, 3 (2 %)	No. 14, 5 (6 %)	n.s.
No opinion	9 (4 %)	5 (4 %)	4 (5 %)	n.s.

*n.s.* non-significant

*p < 0.05 (two-sided), **p < 0.01 (two-sided)

The ranking of 14 strategies as *ideal* ways in the future to bridge the gap between science and policy is shown in Table 5. There is a high level of consistency between the two countries. In slightly varying orders, the top three ranked future strategies for bridging the gap between science and policy were consistent between Chinese and Canadian participants. These strategies were “b. Focus on policy” (China 56 %, Canada 64 %), “i. Science-policy forums” (China 60 %, Canada 55 %), and “c. Policy briefs” (China 53 %, Canada 60 %). There were no significant differences for most future strategies between China and Canada, with the exception of two strategies: “e. Email updates” was rated significantly lower in China than in Canada (7 vs. 22 %), while “g. Conferences” was rated significantly higher by Chinese respondents than Canadian ones (39 vs. 14 %).

**Table 5:** Ranking of 14 strategies by percentage in favor of each strategy with respect to the question “What are some ideal ways that could be used to bridge the gap between science and policy in the future that you would like to see?” in the China-Canada survey 2012

Strategy	China and Canada *n* = 207 (100 %)	China *n* = 121 (100 %)	Canada *n* = 86 (100 %)	*p* value (China vs. Canada)
a. Collaboration in study design	No. 4, 95 (46 %)	No. 6, 53 (44 %)	No. 4, 42 (49 %)	n.s.
b. Focus on policy	No. 1, 123 (59 %)	No. 2, 68 (56 %)	No. 1, 55 (64 %)	n.s.
c. Policy briefs	No. 3, 109 (53 %)	No. 3, 57 (47 %)	No. 2, 52 (60 %)	n.s.
d. Web-based portals	No. 11, 42 (20 %)	No. 11, 19 (16 %)	No. 9, 23 (27 %)	n.s.
e. Email updates	No. 12, 28 (14 %)	No. 14, 9 (7 %)	No. 10, 19 (22 %)	*p* < 0.01**
f. Journal publications	No. 13, 24 (12 %)	No. 12, 18 (15 %)	No. 14, 6 (7 %)	n.s.
g. Conferences	No. 9, 59 (29 %)	No. 7, 47 (39 %)	No. 12, 12 (14 %)	*p* < 0.01**
h. Policy recommendations	No. 5, 83 (40 %)	No. 4, 55 (45 %)	No. 8, 28 (33 %)	n.s.
i. Science-policy forums	No. 2, 120 (58 %)	No. 1, 73 (60 %)	No. 3, 47 (55 %)	n.s.
j. Joint research projects	No. 5, 83 (40 %)	No. 4, 54 (45 %)	No. 7, 29 (34 %)	n.s.
k. Personal contact	No. 8, 65 (31 %)	No. 9, 33 (27 %)	No. 6, 32 (37 %)	n.s.
l. Knowledge brokers	No. 10, 45 (22 %)	No. 10, 26 (21 %)	No. 10, 19 (22 %)	n.s.
m. Collaboration in analysis	No. 7, 71 (34 %)	No. 8, 36 (30 %)	No. 5, 35 (41 %)	n.s.
n. Co-authorship	No. 14, 22 (11 %)	No. 13, 12 (10 %)	No. 12, 10 (12 %)	n.s.
No opinion	13 (6 %)	9 (7 %)	4 (5 %)	n.s.

*n.s.* non-significant

*p < 0.05 (two-sided), **p < 0.01 (two-sided)

Tables 4 and 5 were integrated to look at how strategies were considered differently as current vs. future ideal ways to bridge the gap between science and policy (Table 6). Respondents in China were quite consistent in how they considered the strategies as current vs. ideal ways to bridge the gap between science and policy—both the rankings and the percentages in favor of each strategy as current vs. ideal ways were very close, and none of the differences were significant. For respondents in Canada, however, differences were found in both the rankings and the percentages in favor of each strategy as current vs. ideal ways. The most pronounced changes were observed for “f. Journal publications”, which dropped from No. 4 and 43 % (current) to No. 14 and 7 % (ideal), and for “g. Conferences”, which dropped from No. 3 and 44 % (current) to No. 12 and 14 % (ideal). Other notable differences include: “a. Collaboration in study design”, which rose from No. 9 and 30 % (current) to No. 4 and 49 % (ideal), “c. Policy briefs”, which rose from No. 5 and 42 % (current) to No. 2 and 60 % (ideal), and “m. Collaboration in analysis”, which rose from No. 12 and 24 % (current) to No. 5 and 41 % (ideal).

**Table 6:** Comparison of ranking of 14 strategies by percentage in favor of each strategy as current vs. future ideal ways to bridge the gap between science and policy in the China-Canada survey 2012 (derived from Tables 4 and 5)

	China and Canada *n* = 207 (100 %)			China *n* = 121 (100 %)			Canada *n* = 86 (100 %)		
Strategy	Current	Ideal	*p* value (current vs. ideal)	Current	Ideal	*p* value (current vs. ideal)	Current	Ideal	*p* value (current vs. ideal)
a. Collaboration in study design	No. 7, 77 (37 %)	No. 4, 95 (46 %)	n.s.	No. 7, 51 (42 %)	No. 6, 53 (44 %)	n.s.	No. 9, 26 (30 %)	No. 4, 42 (49 %)	*p* < 0.05*
b. Focus on policy	No. 1, 117 (57 %)	No. 1, 123 (59 %)	n.s.	No. 1, 73 (60 %)	No. 2, 68 (56 %)	n.s.	No. 2, 44 (51 %)	No. 1, 55 (64 %)	n.s.
c. Policy briefs	No. 3, 97 (47 %)	No. 3, 109 (53 %)	n.s.	No. 3, 61 (50 %)	No. 3, 57 (47 %)	n.s.	No. 5, 36 (42 %)	No. 2, 52 (60 %)	*p* < 0.05*
d. Web-based portals	No. 11, 46 (22 %)	No. 11, 42 (20 %)	n.s.	No. 11, 20 (17 %)	No. 11, 19 (16 %)	n.s.	No. 9, 26 (30 %)	No. 9, 23 (27 %)	n.s.
e. Email updates	No. 13, 33 (16 %)	No. 12, 28 (14 %)	n.s.	No. 13, 16 (13 %)	No. 14, 9 (7 %)	n.s.	No. 13, 17 (20 %)	No. 10, 19 (22 %)	n.s.
f. Journal publications	No. 9, 65 (31 %)	No. 13, 24 (12 %)	*p* < 0.01**	No. 10, 28 (23 %)	No. 12, 18 (15 %)	n.s.	No. 4, 37 (43 %)	No. 14, 6 (7 %)	*p* < 0.01**
g. Conferences	No. 3, 97 (47 %)	No. 9, 59 (29 %)	*p* < 0.01**	No. 4, 59 (49 %)	No. 7, 47 (39 %)	n.s.	No. 3, 38 (44 %)	No. 12, 12 (14 %)	*p* < 0.01**
h. Policy recommendations	No. 5, 85 (41 %)	No. 5, 83 (40 %)	n.s.	No. 6, 56 (46 %)	No. 4, 55 (45 %)	n.s.	No. 7, 29 (34 %)	No. 8, 28 (33 %)	n.s.
i. Science-policy forums	No. 2, 112 (54 %)	No. 2, 120 (58 %)	n.s.	No. 2, 65 (54 %)	No. 1, 73 (60 %)	n.s.	No. 1, 47 (55 %)	No. 3, 47 (55 %)	n.s.
j. Joint research projects	No. 5, 85 (41 %)	No. 5, 83 (40 %)	n.s.	No. 5, 58 (48 %)	No. 4, 54 (45 %)	n.s.	No. 8, 27 (31 %)	No. 7, 29 (34 %)	n.s.
k. Personal contact	No. 8, 70 (34 %)	No. 8, 65 (31 %)	n.s.	No. 9, 35 (29 %)	No. 9, 33 (27 %)	n.s.	No. 6, 35 (41 %)	No. 6, 32 (37 %)	n.s.
l. Knowledge brokers	No. 12, 41 (20 %)	No. 10, 45 (22 %)	n.s.	No. 12, 19 (16 %)	No. 10, 26 (21 %)	n.s.	No. 11, 22 (26 %)	No. 10, 19 (22 %)	n.s.
m. Collaboration in analysis	No. 10, 61 (29 %)	No. 7, 71 (34 %)	n.s.	No. 8, 40 (33 %)	No. 8, 36 (30 %)	n.s.	No. 12, 21 (24 %)	No. 5, 35 (41 %)	*p* < 0.05*
n. Co-authorship	No. 14, 8 (4 %)	No. 14, 22 (11 %)	n.s.	No. 14, 3 (2 %)	No. 13, 12 (10 %)	n.s.	No. 14, 5 (6 %)	No. 12, 10 (12 %)	n.s.
No opinion	9 (4 %)	13 (6 %)	n.s.	5 (4 %)	9 (7 %)	n.s.	4 (5 %)	4 (5 %)	n.s.

*n.s.* non-significant

*p < 0.05 (two-sided) **p < 0.01 (two-sided)

**Fig. 1:**
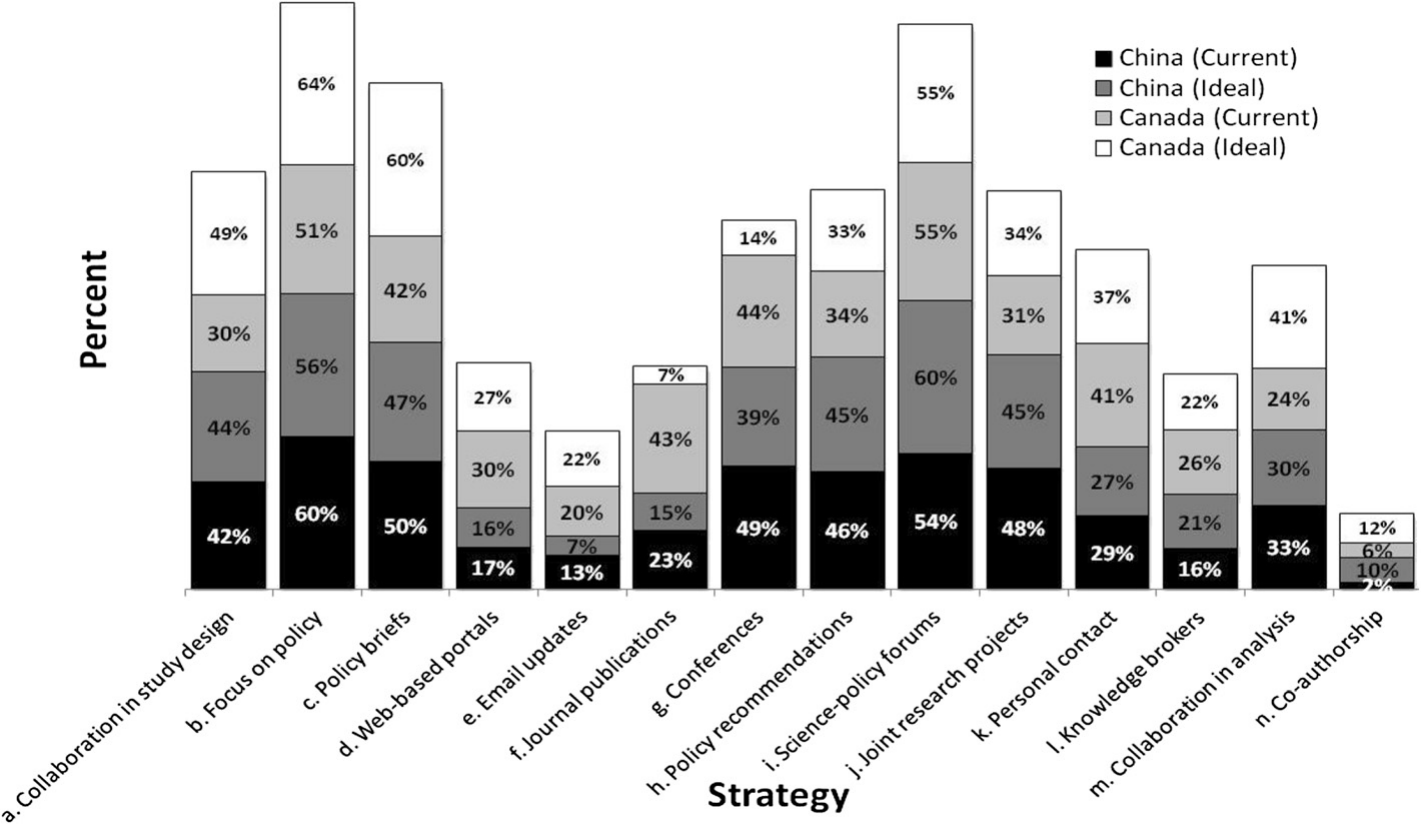
Percentage of Chinese and Canadian participants in favor of each strategy as current and ideal ways used to bridge the gap between and policy

Based on all responses from both China and Canada, and for both current and future ideal ways, the top three overall strategies to bridge the gap between science and policy were: (1) “b. Focus on policy” (58 % or (60 + 56 + 51 + 64 %)/4), (2) “i. Science-policy forums” (56 %), and (3) “c. Policy briefs” (50 %) (Fig. 1).

## Discussion

Caplan coined “two communities” in 1979 to describe the perceived gap between scientists and policy makers [15]. However, research is now considered largely a concept of science-based policy or of negotiations between the scientific community and policy makers [16]. While science profits from society’s growing demand for research, researchers simultaneously face pressure from society’s expectation that science should produce knowledge for evidence-informed policy making. Scientists and policy makers are beginning to join forces to narrow the gap between them [3, 4]. Operating in this gap are many knowledge experts who use research to influence policy and often carry out knowledge transfer, knowledge brokering, and other activities. It is under these changing environments that our China-Canada study was carried out to understand more about the gap and to help speed up the process of bridging it.

China and Canada were selected for this international comparison because these two countries have distinctly different backgrounds in terms of history, culture, and thinking. This maximizes the possibility of finding new thinking and different priorities for bridging the gap between science and policy. Similarities and differences in responses from the two countries strengthen generalizability of the study results to a more global scale.

Participants of this survey were high-ranking scientists and policy makers in the universities and governments with health as a focus of work. They included presidents and professors of universities, and deputy ministers, directors general, and directors of governments. This ensures the inclusion and consideration of opinions at a high level of both scientific evidence producers (scientists) and scientific evidence users (policy makers), in both the scientific research setting (universities) and the policy-making setting (governments).

Among the background characteristics assessed, respondents from China and Canada had a lot in common, especially in terms of their perception of the importance of bridging the gap between science and policy. However, those from China were more likely to be male with many years of experience as scientists, but less as policy makers. Those from Canada tended to be older and more likely to have worked for many years as policy makers. Due to the demographic and professional differences between Chinese and Canadian respondents, a direct comparison of survey responses between the countries should be done with caution. On the other hand, this is part of the objective of the study to benefit from the wide difference in the history, culture, and way of thinking from China and Canada in order to maximize the possibility of finding new ideas.

The major finding in this study is that both Chinese and Canadian high-ranking scientists and policy makers in the universities and governments converged on two priority strategies—focus on policy and science-policy forums—as both current as well as ideal ways to bridge the gap. Our findings taken with other study findings seem to suggest that things have been changing over the years. The literature review published by Innvaer and colleagues in 2002 identifies personal contact between researchers and policy makers to be the most important strategy to facilitate use of research evidence by health policy makers [17]. In our survey conducted in 2012, personal contact has dropped to the eighth current strategy in priority ranking (ninth by China and sixth by Canada). Our finding, however, is consistent with the more recent update of the literature review published by Innvaer’s group, which reports that availability and access to research/improved dissemination, and collaboration between researchers and policymakers, are the top strategies [18].

Conducting research that focuses on policy questions is not a surprising finding, given that this is what bridging the gap between science and policy is about [19, 20]. It has been suggested that evidence-informed policy making should become a “climate” [21], a “general climate” [19], and a “culture” [22]. Science-policy forum is a form of stakeholder policy dialog [23]. In this case, the stakeholders are the scientists who produce the evidence. These forums and dialogs allow interactions between scientists and policy makers, and increase the likelihood of research being used by policy makers [24]. An example is the publication of the 2012 Health-Adjusted Life Expectancy in Canada Report [25]. This report has 77 pages, 6 chapters, and 8 technical appendices. Ten scientists and 10 policy makers were invited to attend two half-day sessions to look at the tables and figures, and derived 25 policy statements (golden nuggets). Other priority strategies identified in our study are well supported in the literature, such as policy briefs [24, 26, 27], collaboration in study designs [18, 28], policy recommendations [27], joint research projects [24], conferences [24], personal contact [24], and collaboration in analysis [18, 28].

In terms of the 14 strategies being currently used, participants from China rated joint research projects more highly than their Canadian counterparts, while those from Canada rated web-based portals and journal publications more highly than those from China. As for the 14 strategies being ideal ways to be used in the future, participants in China rated conferences more favorably than those in Canada, while the reverse was true for email updates. As one of the most wired countries in the world, Canada seems to be more in favor of using information technology than conventional ways to facilitate the science-policy interface.

It is clear from the findings of the study that Canada and China can learn from each other. First, there are similarities in the responses in the two countries which reassure and confirm that they have some good thinking and practices in common. These include focus on policy (conducting research that focuses on policy questions), science-policy forums, and policy briefs, and that conferences are a priority strategy as a current way but not an ideal way in the future. Second, they can learn from the differences in the survey findings in the two countries. In particular, China can find out whether the different opinion among Canadian participants, who are more in favor of using information technology (web-based portals and email updates), and using collaboration in both study design and analysis, and policy briefs as priority ways in the future, means anything for implementation in China. Canada can learn from why Chinese participants are more in favor of joint research projects, and how they set up partnerships between university scientists and government scientists in joint research projects.

It is of interest to look at how the participants from the 2 countries considered the 14 strategies differently as current vs. ideal ways to bridge the gap between science and policy. Respondents in China were quite consistent in their selections of strategies for current vs. ideal ways to bridge the science-policy gap, perhaps indicating a satisfaction with the status quo. Their Canadian colleagues, however, were moving from the more traditional ways, such as journal publications and conferences, to more active and proactive collaboration in study design as well as in analysis. Our study results from Canada are once again consistent with the recent finding of Oliver et al. that collaboration between researchers and policymakers is an important strategy [18].

The usual answer to how to bridge the gap between research and practice or policy is to disseminate scientific findings more efficiently. This focuses on the “back end” or “downstream” (knowledge dissemination) of the knowledge transfer process. But as pointed out by Green, perhaps the question should not be how to get more and better dissemination and implementation of the existing science to practitioners and policymakers, but instead, how to ask the right questions in the first place and, in turn, how to get better adaptation of the research practices into the real world [29]. If so, the focus needs to shift to the “front end” or “upstream” (knowledge generation), which is the collaboration between scientists and policy makers in study design and in analysis. These are the stages before even knowledge products are obtained.

A limitation of this study is the low response rates (30.0 % for China, 15.9 % for Canada), given the nature of the respondents. Low response rate is a troubling trend in recent years [30, 31]. In our study, the respondents were a very special group (high-ranking scientists and officials in universities and governments). Many of the recommendations mentioned by Edwards et al. [32] to increase response to electronic surveys (such as non-monetary incentives, lottery, and statements that others had responded) were not expected to work among our study participants, as incentives and lottery are not allowed for government participants, and high-ranking officials seldom care whether others have responded. However, we had used shorter e-Questionnaires, interesting topics, a white background, giving a deadline, and most importantly, repeated follow-up email reminders [31–33]. Results of this study should be considered an opinion survey of a more motivated group of scientists and policy makers who volunteered their suggestions and comments on how to better bridge the gap between them. A second limitation is that, through job title screening for high-ranking scientists and officials, participants would likely have involvement in evidence-informed health policy, but there is no guarantee. For example, there could be “high-ranking” professors in the universities who focus on the area of health, but their research could not be linked to policy at all. To confirm policy research experience would necessitate a two-phase survey which would make the study too complex to conduct, or asking at the beginning of the survey “are you involved in policy research” and then deleting individuals not working in policy research from the survey. Deletion of non-policy research individuals might introduce a bias as those individuals might have good innovative insights into how their research might be used for policy. On the other hand, the effect of the dilution of the study sample by non-policy research individuals is not expected to cause any bias (systematic error) but will make the findings more conservative (less likely to find differences).

## Conclusions

Our international study has confirmed a number of previously reported priority strategies to bridge the gap between science and policy. These include: focus on policy, science-policy forums, policy briefs, collaboration in study design, policy recommendations, joint research projects, conferences, personal contact, and collaboration in analysis.

More importantly, our international study has contributed to the future work on evidence-based policy making by comparing the responses from China and Canada, and the responses for current and ideal ways for the future. Our findings that participants from China are significantly more in favor of joint research projects, and those from Canada are more in favor of web-based portals than their counterparts, are interesting and should be monitored in the future. Additionally, the views from Canada that collaboration in study design and analysis will increase in importance as an ideal way in future, while journal publications and conferences will be less important, could signal a change in shifting the focus from the “back end” or “downstream” (knowledge dissemination) of the knowledge transfer process to the “front end” or “upstream” (knowledge generation). Comparison of our 2012 study with the 2002 study by Innvaer et al. [17] show that personal contact previously identified as the top strategy is no longer considered very important in our participants. All these show that the concept and strategies of bridging the gap between science and policy are not static, but varying in space and evolving over time.

## Ethics approval

The study obtained ethics approval from the research ethics boards of the Medical College of Shantou University, China and the Faculty of Medicine of University of Ottawa, Canada.

**Table:** 

Policy Points • The international study of high-ranking scientists and policy makers who have a senior position in universities and governments in the health sector in China and Canada identified a number of common strategies, as well as country-specific strategies to bridge the gap between science and policy. • The top strategies selected by respondents included: focus on policy (conducting research that focuses on policy questions), science-policy forums, and policy briefs. • Our study detected a possible shift in the thinking from the current focus on the “back end” or “downstream” (knowledge dissemination) of the knowledge transfer process to a future ideal focus on the “front end” or “upstream” (knowledge generation).	
